# Fussing

**Published:** 1874-11

**Authors:** 


### FUSSING.

Some people are forever fussing about their health; a great part of their occupation seems to be feeling about to see if they have not some "symptom" to speak about or complain of, and no sooner do they find one than they begin to "take something;" many unfortunates "take a drink," and before they know it are drunkards; others take physic until their stomach is a well-appointed apothecary's shop. They shake their heads to see if they do not have the headache; they poke their sides to see if there is not a pain there; they feel the pulse to find out July fever, and are looking at their tongue a dozen times a day to discover some approach to biliousness. As soon as they discover a symptom, they run their eyes along the columns of a newspaper to see if there is not something advertised good to take—their money out of their pockets. With the uniform result, they are always

TAKING SOMETHING, and are always sick; always bothering others with a long detail of their sufferings, and often get so that they talk about nothing else.

Very few persons in perfect health fail to have some little bodily derangement or irregularity in any single week of their life; but knowing they are well, the mind does not dwell upon it, goes out towards something else, and that is the last of it. It should always be borne in mind that a large share of our little aches and pains would pass off about as soon by letting them alone as by doing or taking something, and the more we "take" the greater the necessity of "taking."

